# Identifying and ranking of the main organizational resilience indicators in the hospital during the COVID-19 pandemic: A study using fuzzy Delphi technique (FDT) and fuzzy analytical hierarchy process (FAHP)

**DOI:** 10.1016/j.heliyon.2024.e27241

**Published:** 2024-02-28

**Authors:** Mojtaba Haghighat, Seyed Mahdi Mousavi, Mahsa jahadi naeini

**Affiliations:** a Behbahan Faculty of Medical Sciences, Behbahan, Iran; b Student Research Committee, Department of Occupational Health and Safety Engineering, School of Health, Isfahan University of Medical Sciences, Isfahan, Iran

**Keywords:** Hospitals, COVID-19, Resilience, Emergencies, Delphi technique, FAHP

## Abstract

Resilience in a hospital is referred to as the capability to withstand and respond to disasters while maintaining critical functions. The current study aims to identify and prioritize the defining indicators of organizational resilience in dealing with the critical conditions of the COVID-19 pandemic. First, a literature review and semi-structured interviews with experts were performed for the identification of the primary indicators affecting the hospital's organizational resilience in critical conditions caused by the COVID-19 disease. Next, the fuzzy Delphi technique (FDT) was used to determine the effective and final indicators with a 70% agreement level. Finally, the indicators were weighted and prioritized using the FAHP method. The results shows that three indicators of leadership and management (NW = 0.092), preparedness (NW = 0.080), and learning from previous experiences (NW = 0.078) had the greatest impact on the organizational resilience of the hospital, respectively. Three indicators of logistics support, fallibility culture, creativity, and innovation with the final weights of 0.56, 0.054, and 0.053 gained the least importance, respectively. It can be concluded that a higher level of leadership and management, preparedness, and learning from previous experiences in the conditions of the COVID-19 pandemic can help control this crisis.

## 1 Introduction

The occurrence of accidents and disasters affects the lives of humans around the world and hampers the development of nations and societies [1]. Accidents can be classified as natural accidents (such as earthquakes, floods, storms, and military events) and artificial accidents (such as biological events and radioactive material leakage) [2]. Epidemic viral-infectious diseases can be considered as a magnificent health challenge [3]. The first case of COVID-19 was reported in December 2019 in Wuhan, China. The rapid transmission of this virus led to the diagnosis of more than a million patients within a few months [4,5]. A significant increase in the number of infected patients creates a difficult condition for hospitals as the front lines of the medical system [6]. The role of hospitals and healthcare centers is very sensitive when accidents and disasters occur. Fast, optimal, and timely health-treatment services can reduce mortality and increase the number of survivors [[7], [8], [9]]. Therefore, it is very crucial to provide strategies for improving resilience in health and treatment centers [10]. In order to manage biological crises in hospitals, the identification of what it is found as the challenges for hospitals, along with solutions and measures, can be very effective [11]. In addition, in term of the design, the hospital must endure least damage during natural or man-made disasters and accidents. Also, their capacity and response to the health and medical needs of the community should be quickly increased [12]. Hospitals must have high organizational resilience to deal with such crises and disasters. Resilience refers to the hospital's capability to withstand and respond to disasters so that it can maintain its defined structure and perform its routine activities [13,14]. Some researchers presented a framework of resilience and methods of its measurement. Olu provided a resilient health system, as a conceptual framework for strengthening risk management of public health disaster [15]. Cimellaroin et al. mentioned the three important factors affecting hospital disaster resilience including cooperation and training management, resources and equipment capability, and structural and organizational operating procedures [16]. Zhong et al. performed a study on the resilience tool of hospitals in China and identified four final factors including emergency response capability, disaster management mechanism, hospital infrastructure safety, and disaster sources [17]. Anderson et al. explained a method for development of indicators of organizational resilience by establishing a balance between organizational processes and structures. They introduced risk awareness and cooperation as the main indicators of organizational resilience [18]. The results of the previous studies show that the determination of the indicators affecting resilience can result in a better analysis and more consistent planning in achieving a resilient hospital during epidemics and pandemics [19,20].

The Delphi technique can be employed in qualitative research with an exploratory aspect for the identification of the fundamental elements of a phenomenon [21]. This technique is a structured process for gathering information and obtaining group consensus. The main goal of this method is the consensus of a group of experts with various opinions. Researchers can identify the factors and develop a framework for their diagnosis using this process [22]. Given that experts’ opinions are highly subjective, it is recommended that fuzzy numbers are applied [23]. The fuzzy Delphi technique was first introduced in 1988 by Kaufman and Gupta. In this technique, the traditional Delphi and fuzzy theory have been combined [24]. The Delphi method has been applied in previous studies to identify the criteria required for the selection of sound control solutions in various industries and to determine safety indicators required for the evaluation of crisis management. Thus, this method can be used in various fields of occupational safety and health [25,26]. Considering that the importance of all identified indicators is not equal, the use of multi-criteria decision-making (MCDM) techniques can be helpful [27]. These techniques reveal the importance and weight of the criteria. One of the most common multi-criteria decision-making (MCDM) methods is the Analytical Hierarchy Process (AHP). This method was developed by Saati in 1980. It is known as a powerful and flexible technique for solving complex problems [28]. The AHP method combines objective and subjective evaluations with an integrated structure based on scales of paired comparisons, and it organizes the essential aspects of a problem with a hierarchical format [29]. In this method, because of the uncertainty of pairwise comparisons, decision-makers have difficulty expressing their opinion on superiority of elements. in order to solve this problem, fuzzy logic is used in the analytical hierarchy process method. In FAHP method, experts are asked to compare the elements and express their relative importance using fuzzy numbers [30]. Given the importance of the pandemic crisis, more studies are required to investigate the various indicators affecting organizational resilience. Therefore, the present study aims to identify and prioritize the important indicators of organizational resilience in a hospital during the COVID-19 pandemic.

## 2 Materials and methods

### 2.1 Hospital selection

The present study was conducted in one of the hospitals located in the southwest of Iran. With 300 staff members, this hospital was designated as a special center for the treatment and care of COVID-19 patients. Prior to the outbreak of the COVID-19, this hospital had covered a region with a population of around 180,000 people, whereas, this number soared to 200,000 during COVID-19. As the sole COVID-19 referral center, this hospital was also located in an area with a staggering number of Covid-19 death rate. Various factors such as being understaffed, lack of sufficient space, increase in staff working hours, etc. left this center with numerous challenges in providing services. In order for service provider centers such as hospitals to function normally under crises, resilience is of a great importance. Accordingly, identifying indicators related to resilience in this hospital is one of the necessities that can provide proper identification of the strengths and weaknesses of the center in the face of the COVID-19 crisis.

### 2.2 Study design

This descriptive-analytical study aimed to identify and prioritize the main indicators of organizational resilience in hospitals during the COVID-19 pandemic using fuzzy Delphi and FAHP methods. First, a literature review and semi-structured interviews were performed with the aim of identification of the primary indicators affecting the hospital's organizational resilience in critical conditions caused by the COVID-19 disease. Second, the fuzzy Delphi technique (FDT) was applied to determine the final indicators. Finally, the indicators were weighed and prioritized using the FAHP method. A detailed three-step description of the study is provided as follows.

### 2.3 Identifying resilience indicators related to hospitals

At this stage, the free review was done in valid databases such as Web of Science, Scopus, Google Scholar, SID, and Magiran using the keywords of resilience, organizational resilience, resilience engineering, resilient organizations, technical resilience, resilience indicators, measuring of resilience, resilience in crises, resilience in hospitals, resilience and major accidents, adaptive capacity, crisis, emergency management, and emergency preparedness. As inclusion criteria, quantitative and qualitative studies on hospital resilience in the field of crisis management were selected. In the next step, the titles were examined, duplicates and unrelated items were removed, and the abstracts of the remaining articles were studied. A total of 40 articles related to the topic were selected. Finally, the full texts of selected studies were received, and the primary indicators affecting organizational resilience were identified.

Moreover, in addition to free review, a semi-structured interview was performed with the head of the hospital, hospital managers, hospital supervisors and academic staff in order to identify indicators affecting resilience in the hospital. For this purpose, the subjects were asked to answer a question (which indicators can affect the hospital's resilience). The interviews were also recorded for more detailed analysis. In this research, due to the conditions created by the COVID-19, face-to-face and virtual interviews were conducted through communication platforms such as Skype and WhatsApp. A total of 40 semi-structured interviews were conducted. A total of 15 initial indicators affecting resilience obtained from free review and interviews were identified in this stage and entered the next stage in order to determine the final indicators.

### 2.4 The fuzzy-Delphi technique

**Fig. 1:**
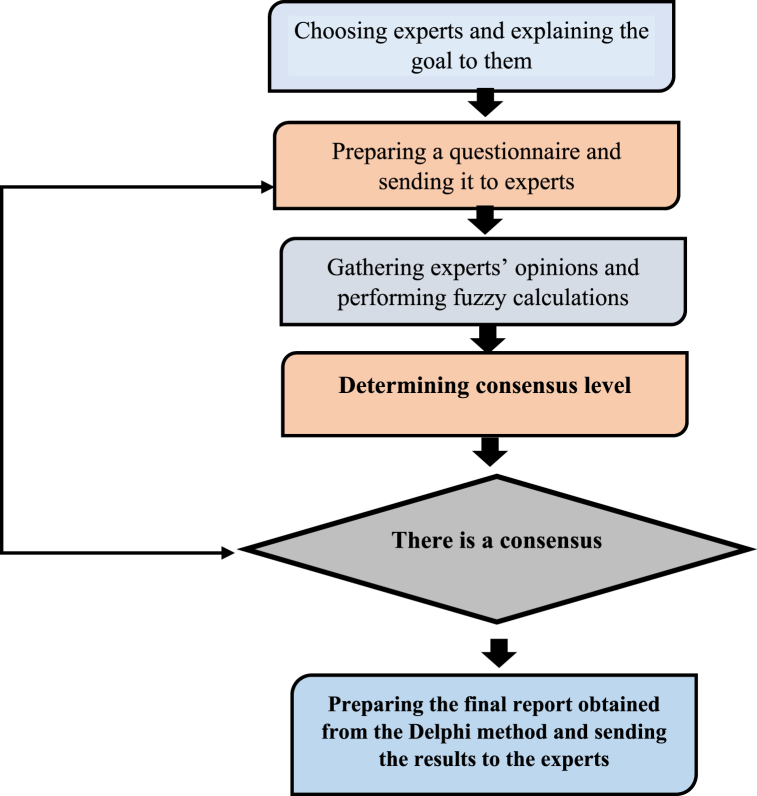
Steps of emplaning fuzzy Delphi Technique.

The fuzzy Delphi method presented as follows in Fig. 1 [26,31].

#### 2.4.1 Selection of expert panel

First, the qualified experts were identified and the sampling was done according to convenience sampling method. Therefore, those who were willing to cooperate and also sought to familiarize themselves with the concepts of resilience in the hospital were included in the expert team as a sample size. A total of 40 people were selected as a team of experts to perform fuzzy Delphi method and FAHP. Then, they were briefed on the purposes and steps of the study. The final panel consisted of the hospital president, hospital managers, hospital supervisors, and academic staff. The average age of participating experts was 38.87 ± 6.1. The minimum and maximum age of the experts was 29 and 50 years, respectively, and the average work experience of people was 12 ± 4 years. Table 1 reports the detail of demographic characteristics of the expert panel.

**Table 1:** Demographic characteristics of the expert panel (N = 40).

Variables		Frequency	Percent (%)
Age (year)	Less than 30	5	12.5
	31 to 35	7	17.5
	36 to 40	13	32.5
	41 to 45	8	20
	More than 46	7	17.5
sex	Male	18	45
	Female	22	55
Education level	Bachelor of Science	15	37.5
	Master of Science	10	25
	PhD	15	37.5
Marital status	Single	10	25
	Married	30	75
Job experience (year)	1–5	5	12.5
	6–10	10	25
	11–15	15	37.5
	More than 15	10	25
Job position	Hospital managers	10	25
	Hospital supervisors	20	50
	Academic staff	10	25

#### 2.4.2 Collecting experts’ opinions

In this step, similar to the traditional Delphi method, the experts’ opinions were collected. In this method, linguistic variables were applied to design a questionnaire and gather experts' opinions (see Table 2).

**Table 2:** Linguistic expression and importance score.

Linguistic expression	Importance score
Very low	1
Low	2
Medium	3
High	4
Very high	5

#### 2.4.3 Calculating the fuzzy value of each question

The linguistic expression of the criterion j from the viewpoint of an expert with the number i among persons is Wij = (aij, bij, cij). The value of j is equal to j = 1, 2, 3, …, m and the value of i is equal to i = 1,2,3, …, n. In this step, the fuzzy value of criterion j was calculated by the following equation, which is equal to Wj= (aj, bj, cj).

(1) aj=min{aij}

(2) $$\text{bj}=\frac{1}{n}\sum_{i=1}^{n}bj$$

(3) cj=max{bij}

#### 2.4.4 Converting the fuzzy value

In this step, the obtained fuzzy value for each of the questions was converted to a de-fuzzified value. The following equation was used for this purpose.

(4) $$\text{Sj}=\frac{aj+bj+cj}{3}\;i=1,2,\ldots m$$

#### 2.4.5 Examining experts’ agreement

In this step, the acceptable level of consensus or agreement of experts was 70 percent. This level of agreement was determined based on previous studies that used the fuzzy Delphi method [26,30].

### 2.5 Fuzzy hierarchy analysis methods (FAHP)

Chang presented a method (in 1992) to extend the fuzzy hierarchical analysis process [32]. In the present study, this method was used to calculate the final weight of the indicators. The steps were as follows [30,33].

#### 2.5.1 Defining fuzzy numbers

In this step, the linguistic words of experts were converted to fuzzy numbers, described in Table 3. The Fuzzy analytical hierarchical process was performed based on their criteria and opinions.

**Table 3:** Linguistic words and their synonymous triangular fuzzy numbers.

Linguistic terms	Fuzzy number scale	Fuzzy numbers
Equally important	1	(1,1,1)
Equally important to slightly more important	2	(1,2,3)
Slightly more important	3	(2,3,4)
Slightly more important to more important	4	(3,4,5)
More important	5	(4,5,6)
More important to much more important	6	(5,6,7)
Much more important	7	(6,7,8)
Much more important to extremely more important	8	(7,8,9)
Extremely more important	9	(8,9,10)

#### 2.5.2 Forming paired comparison matrix (A) using fuzzy numbers

The paired comparison was performed by the decision matrix, shown in Eq. (5).

(5) $$\tilde{A}=\left[\begin{array}{c} \begin{array}{c} 1\\ M_{21} \end{array}\\ \vdots \\ M_{n1} \end{array}\begin{array}{c} \begin{array}{c} M_{12}\\ 1 \end{array}\\ \vdots \\ M_{n2} \end{array}\begin{array}{c} \begin{array}{c} \cdots \\ \cdots \end{array}\\ \ddots \\ \cdots \end{array}\begin{array}{c} \begin{array}{c} M_{1n}\\ M_{2n} \end{array}\\ \vdots \\ 1 \end{array}\right]$$

#### 2.5.3 Calculating Si

Si is the triangular fuzzy number related to the relative weight of each criterion which is calculated by Eq. (6).

(6) $$S_{i}=\sum_{j=1}^{m}M_{gi}^{J}\times {\left[\sum_{i=1}^{n}\sum_{j=1}^{m}M_{gi}^{J}\right]}^{-1}$$

In this equation, i, j and $M_{gi}^{j}$ are the column number, row number, and fuzzy numbers of the paired matrix, respectively. $\sum_{j=1}^{m}M_{gi}^{J}$ , $\sum_{i=1}^{n}\sum_{j=1}^{m}M_{gi}^{i}$ and ${\left[\sum_{i=1}^{n}\sum_{j=1}^{m}M_{gi}^{i}\right]}^{-1}$ are computed using the following equations.

(7) $$\sum_{j=1}^{m}M_{gi}^{j}=\left(\sum_{j=1}^{m}l_{j},\sum_{j=1}^{m}m_{j},\sum_{j=1}^{m}u_{j}\right)$$

(8) $$\sum_{i=1}^{n}\sum_{j=1}^{m}M_{gi}^{j}=\left(\sum_{i=1}^{m}l_{i},\sum_{i=1}^{m}m_{i},\sum_{i=1}^{m}u_{i}\right)$$

(9) $${\left[\sum_{i=1}^{n}\sum_{j=1}^{m}M_{gi}^{j}\right]}^{-1}=\left(\frac{1}{\sum_{i=1}^{m}u_{i}},\frac{1}{\sum_{i=1}^{m}m_{i}},\frac{1}{\sum_{i=1}^{m}l_{i}}\right)$$

#### 2.5.4 Calculating possibility degree

If S_1_ = (l_1_, m_1_, u_1_) and S_2_ = (l_2_, m_2_, u_2_) are two triangular fuzzy numbers, the degree of possibility of S_2_ ≥ S_1_ is defined by the following equations.

(10) $$V\;\left(S_{2}\geq S_{1}\right)=hgt\;\left(S_{2}\cap S_{1}\right)=\mu_{S_{1}}\left(d\right)=\left\{ \begin{array}{c} 1\;if\;m_{2}\geq m_{1}\\ 0\;if\;l_{1}\geq u_{2}\\ \frac{l_{1}-u_{1}}{\left(m_{2}-u_{2}\right)-\;\left(m_{1}-l_{1}\right)\;}\;otherwise \end{array}\right.$$

On the other hand, the possibility degree of a triangular fuzzy number relative to k triangular fuzzy numbers was obtained by the following equations.

(11) $$V\;\left(S_{2}\geq S_{1\;},S_{2},\ldots ,S_{K}\right)=V\;\left[\left(S\geq S_{1}\right)\;and\;\left(S\geq S_{2}\right)\;and\ldots and\;\left(S\geq S_{k}\right)\right]=\mathrm{Min}\;V\;\left(S\geq S_{i}\right).\;i=1.\;2.3.\;\ldots .\;k$$

#### 2.5.5 Calculating criteria weight

Equation (8) was used for computing the weight vector of criteria in the paired matrix.

(12) $$d^{\prime }\left(A_{i}\right)=\mathrm{Min}\;V\;\left(S_{i}\geq S_{k}\right)\;k=1.\;2.\;\ldots .\;n\;.\;k\neq i$$

Therefore, the non-normal weight vector will be as follows:

(13) $$W^{\prime }={\left(d^{\prime }\left(A_{1}\right).\;d^{\prime }\left(A_{2}\right).\;\ldots .d^{\prime }\left(A_{n}\right)\right)}^{T}\;A_{i\;}\left(i=1.\;2.\;\ldots .\;n\right)$$

#### 2.5.6 Calculating normal weight

To calculate the normal weight, the non-normal weight vector of the previous step was normalized by Eq. (10).

(14) $$W={\left(d\left(A_{1}\right).\;d\left(A_{2}\right).\;\ldots .d\left(A_{n}\right)\right)}^{T}$$

As well as, the geometric mean value was used to combine experts’ opinions. It was computed by Eq. (11).

(15) aij=(∏K=1KAijk)1KK=1.2.….K

#### 2.5.7 Calculating consistency index

In this step, the consistency index was calculated using the Gogus and Boucher method to ensure the reliability of the results obtained [34]. The results showed that the consistency index of all matrices was less than 0.1. Therefore, the results were considered reliable.

### 2.6 Data analysis

MATLAB software (version 2018a) was used for calculating and analyzing data.

## 3 Result

The results showed that all identified indicators were appropriate. The experts' agreement for all criteria was higher than 70 percent. Table 4 represents the results of the fuzzy-Delphi technique.

**Table 4:** The results of the fuzzy-Delphi technique.

Resilience Indictors in Hospitals (symbol)	Importance spectrum					Fuzzy value aggregation			Defuzzied value	Consensus Percentage
	Very low (1)	Low (2)	Medium (3)	High (4)	Very high (5)	L	M	U		
Logistics support	0	3	2	31	2	2	3.65	5	3.57	0.775
Adaptive capacity	0	0	3	8	29	3	4.65	5	4.32	0.725
Planning strategy and goal setting	0	0	7	28	5	3	3.25	4	3.37	0.700
Responsibility	1	2	3	29	5	4	3.15	5	3.82	0.725
Preparedness	0	0	2	3	30	3	4.20	5	4.1	0.750
Resources (equipment and sufficient personnel)	0	1	5	28	6	2	3.95	5	3.72	0.700
Communication and teamwork	2	2	1	29	6	1	3.87	5	3.43	0.725
Effective public participation	0	2	3	5	30	2	4.57	5	4.03	0.750
Awareness of the situation	2	2	5	28	3	1	3	5	3	0.700
Leadership and management	0	0	5	5	30	3	4.62	5	4.31	0.750
Creativity and innovation	0	2	5	31	2	1	3.82	5	3.41	0.775
Fallibility culture	2	3	1	28	6	2	3.82	5	3.66	0.700
Learning from previous experiences	0	2	2	6	30	3	4.6	5	4.3	0.750
Education	0	0	3	30	7	3	4.1	5	4.05	0.750
Inter-organizational coordination	0	4	6	29	1	2	3.67	5	3.58	0.725

The results of FAHP in Table 5 indicated that three indicators of leadership and management (NW = 0.092), preparedness (NW = 0.080), and learning from previous experiences (NW = 0.078) had the greatest impact on the organizational resilience of the hospital, respectively. Also, three indicators of logistics support, fallibility culture, and creativity and innovation with the final weights of 0.56, 0.054, and 0.053 had the least importance, respectively.

**Table 5:** The results of the Fuzzy Hierarchy Analysis methods (FAHP).

Resilience indicator	Fuzzy weight			Relative weight (RW)	Normal weight (NW)
	L	M	U		
Leadership and management	0.061	0.631	2.117	3.44	0.092
Preparedness	0.091	0.561	1.782	2.995	0.080
Learning from previous experiences	0.077	0.432	1.987	2.928	0.078
Adaptive capacity	0.091	0.432	1.798	2.753	0.074
Effective public participation	0.049	0.432	1.781	2.694	0.072
Awareness of the situation	0.112	0.422	1.630	2.585	0.069
Education	0.046	0.421	1.631	2.519	0.067
Planning strategy and goal setting	0.049	0.211	2.011	2.482	0.066
Responsibility	0.071	0.322	1.562	2.277	0.061
Resources (equipment and sufficient personnel)	0.036	0.321	1.578	2.256	0.060
Communication and teamwork	0.036	0.521	1.113	2.191	0.059
Inter-organizational coordination	0.076	0.142	1.812	2.172	0.058
Logistics support	0.081	0.421	1.186	2.109	0.056
Fallibility culture	0.025	0.321	1.371	2.038	0.054
Creativity and innovation	0.061	0.149	1.637	1.996	0.053

## 4 Discussion

In this study, 15 indicators were identified as the factors affecting the organizational resilience of the hospital during the COVID-19 pandemic. The results showed that leadership and management, preparedness, and learning from previous experiences were the most important indicators of organizational resilience in the hospital, respectively. Therefore, the mentioned factors play a defining role in creating and maintaining hospital resilience. The management and leadership behavior in any organization influences the thoughts and perception of employees as it keeps them motivated and guided [35,36]. Hence, the role of management and leadership style should be taken well into account to improve hospital resilience. The results of the studies performed by Jafari et al. and mahmoudi et al. showed that the most important indicator in organizational resilience is leadership and management, which is consistent with the findings of the present study [37,38]. Thus, a hospital or an organization will be resilient if it is led by strong leadership and management teams. Preparedness is defined as the prediction of unexpected events and the capability of timely and appropriate response. In a resilient organization, possible problems are predicted and necessary instructions, emergency response maneuvers, and practical exercises are executed. Davids et al. and Sunindijo et al. have introduced preparedness as one of the most important indicators in organizational resilience. The results of the present study showed that the preparedness with the final weight of 0.080 was the second important indicators of organizational resilience in the hospital, which is consistent with the findings of a study performed by Samsuddin et al. in Malaysian hospitals [39]. Zarrin and Azadeh also obtained similar results and concluded that preparedness and management commitment in emergencies are the two main components to determine the resilience level [40]. Based on the results of the present study, the indicator of learning from previous experiences, with a weight of 0.078, ranked as the third most important indicator of organizational resilience in the hospital. If the management has insufficient knowledge on the technical issues of resilience, serious decisions and actions are improbable. The results of a study performed by Billings et al., in 2021 showed that learning from previous experiences in dealing with crises is a very important item, which is in line with the findings of the present study. Hence, hospital managers should create a system for recording and analyzing the crises and disasters occurred in the organization. This information can be used for training of employees. The managers must plan ahead and have the foresight to avoid incidences, however, they should also accept the fact that crises and incidents may happen in an organization. Managers should avoid the strict and rigid controls and increase the organizational flexibility. Agile organizations with a flexible structure can be better adapted to environmental changes [41,42]. In the present study, the adaptability capacity with the final weight of 0.078 stood the fourth priority among indicators. Furthermore, several studies have introduced the factors of weak management and communication, structural problems, and inadequate budget as the most important problems of hospitals during a crisis, which is consistent with the findings of the present study. In the present study, effective public participation with final weight of 0.072 was recognized as another important indicator. Public association during crises, such as the COVID-19, can affect the transmission chain. The results of a study conducted by Farida et al. showed that the high level of public association plays an important role in reducing the infected people and the death rate due to COVID-19. The results of another study also indicated the important role of public association in controlling the COVID-19 disease. In addition, increase of awareness on this unknown disease can be helpful in public association [43,44]. Eventually,the education indicator in the present study had final weight of 0.067. The role of education, as an important indicator in organizational resilience, has been confirmed in several studies. To increase the knowledge on organizational resilience in the hospital, the use of educational models such as the health belief model (IBM) has been recommended by Zhou et al. [45].

## 5 Methodological limits

The present study was conducted as an example in just one hospital and with a limited number of experts. Considering that several indicators have an effect on resilience, it is recommended that the future studies be conducted in multiple hospitals or similar healthcare environments with a larger panel expert. Another limitation in this study is the internal relationships between organizational resilience indicators, which were ignored. Hence, it is suggested that the internal relationship between the indicators be determined using the DEMATEL technique and the ANP method in future studies. Also, the combination of the fuzzy Delphi technique (FDT) and FAHP techniques can be used to identify and prioritize the resilience indicators in other organizations.

## 6 Conclusion

The results of this study provide helpful information for hospital managers to plan and improve the resilience of hospitals during crises, such as the COVID-19 pandemic. Moreover, the strengths of this study were the identification and prioritization of indicators affecting resilience in the hospital under the conditions of the COVID-19 pandemic. This study proposed the use of FDT-FAHP for identifying and ranking organizational resilience indicators in hospitals during the COVID-19 pandemic: the results showed that the most important indicators of resilience were leadership and management, preparedness and learning, and learning from previous experiences, respectively.

## Ethics statement

Informed consent was obtained from all participants with approval by the ethics committee of Behbahan University of Medical Sciences (code number IR.BHN.REC.1401.024).

## Funding statement

This research did not receive any specific grant from funding agencies in the public, commercial, or not-for-profit sectors.

## CRediT authorship contribution statement

**Mojtaba Haghighat:** Writing – review & editing. **SeyedMahdi mousavi:** Formal analysis. **Mahsa jahadi naeini:** Writing – original draft, Data curation.

## Declaration of competing interest

The authors declare that they have no known competing financial interests or personal relationships that could have appeared to influence the work reported in this paper.
