# Supplementary material for: Identifying and ranking of the main organizational resilience indicators in the hospital during the COVID-19 pandemic: A study using fuzzy Delphi technique (FDT) and fuzzy analytical hierarchy process (FAHP)
Source: Heliyon. 2024 Feb 28;10(5):e27241. doi: 10.1016/j.heliyon.2024.e27241 (PMC10915563; doi:10.1016/j.heliyon.2024.e27241)
Supplement: Multimedia component 1 [file mmc1.pdf]

Delphi fuzzy Questionnaire

Dear expert

The following questionnaire was prepared in order to conduct a research entitled " Application of the Fuzzy Delphi Technique (FDT) and fuzzy Analytical Hierarchy Process (FAHP) for the identifying and ranking main organizational resilience indicators in hospitals during the COVID 19 pandemic ". your answer to these questions will help us in completing the relevant information and analysis. It should be noted that the respondent's name and personal will be kept confidential. Thank you for your cooperation.

number	Questions	Importance score				
		Very little importance (1)	Little importance (2)	Medium importance (3)	Great importance (4)	Very important (5)
1	based on your opinion, how much does the **"logistics support"** indicator play a role in the organizational resilience of the hospital?					
2	based on your opinion, how much does the **"Adaptive capacity"** indicator play a role in the organizational resilience of the hospital?					
3	based on your opinion, how much does the **"Planning strategy and goal setting "**indicator play a role in the organizational resilience of the hospital?					
4	based on your opinion, how much does the **"Responsibility"** indicator play a role in the organizational resilience of the hospital?					
5	based on your opinion, how much does the **"Readiness"** indicator play a role in the organizational resilience of the hospital?					
6	based on your opinion, how much does the **"Resources"** indicator play a role in the organizational resilience of the hospital?					
7	based on your opinion, how much does the **"Communication and teamwork"** indicator play a role in the organizational resilience of the hospital?					
8	based on your opinion, how much does the **"Effective public participation"** indicator play a role in the organizational resilience of the hospital?					
9	based on your opinion, how much does the **"Awareness of the situation"** indicator play a role in the organizational resilience of the hospital?					
10	based on your opinion, how much does the **"Leadership and management"** indicator play a role in the organizational resilience of the hospital?					
11	based on your opinion, how much does the **"Creativity and innovation indicator"** play a role in the organizational resilience of the hospital?					
12	based on your opinion, how much does the **"Fallibility culture "**indicator play a role in the organizational resilience of the hospital?					
13	based on your opinion, how much does the **"Learning from previous experiences "**indicator play a role in the organizational resilience of the hospital?					
14	based on your opinion, how much does the **"Education"** indicator play a role in the organizational resilience of the hospital?					
15	based on your opinion, how much does the **"Inter-organizational coordination"** indicator play a role in the organizational resilience of the hospital?					
