# Supplementary material for: Identifying and ranking of the main organizational resilience indicators in the hospital during the COVID-19 pandemic: A study using fuzzy Delphi technique (FDT) and fuzzy analytical hierarchy process (FAHP)
Source: Heliyon. 2024 Feb 28;10(5):e27241. doi: 10.1016/j.heliyon.2024.e27241 (PMC10915563; doi:10.1016/j.heliyon.2024.e27241)
Supplement: Multimedia component 2 [file mmc2.pdf]

Paired comparison questionnaire (FAHP questionnaire)

Dear expert

The following paired comparison questionnaire is related to a part of the research study titled: " Application of the Fuzzy Delphi Technique (FDT) and fuzzy Analytical Hierarchy Process (FAHP) for the identifying and ranking main organizational resilience indicators in hospitals during the COVID 19 pandemic ". Please compare and score the importance of hospital resilience indicators two by two according to table 1.

Table 1: Linguistic scale and its synonymous

Linguistic terms	Fuzzy number scale
Equally importance	1
Equal importance to slightly more	2
A little more importance	3
A little more importance to more importance	4
More important	5
More important to much more important	6
Much more important	7
Much more important to absolute important	8
Absolute important	9

number 1 means the two causes are equally important. As the numerical value moves towards the number 9, its importance increases compared to the opposite cause.

Indicator A	9	8	7	6	5	4	3	2	1	2	3	4	5	6	7	8	9	Indicator B
Logistics support																		Adaptive capacity
Logistics support																		Planning strategy and goal setting
Logistics support																		Responsibility
Logistics support																		Readiness
Logistics support																		Resources
Logistics support																		Communication and teamwork
Logistics support																		Effective public participation
Logistics support																		Awareness of the situation
Logistics support																		Leadership and management
Logistics support																		Creativity and innovation
Logistics support																		Fallibility culture
Logistics support																		Learning from previous experiences
Logistics support																		Education
Logistics support																		Inter-organizational coordination
**Indicator A**	**9**	**8**	**7**	**6**	**5**	**4**	**3**	**2**	**1**	**2**	**3**	**4**	**5**	**6**	**7**	**8**	**9**	**Indicator B**
Adaptive capacity																		Planning strategy and goal setting
Adaptive capacity																		Responsibility
Adaptive capacity																		Readiness
Adaptive capacity																		Resources
Adaptive capacity																		Communication and teamwork
Adaptive capacity																		Effective public participation
Adaptive capacity																		Awareness of the situation
Adaptive capacity																		Leadership and management
Adaptive capacity																		Creativity and innovation
Adaptive capacity																		Fallibility culture
Adaptive capacity																		Learning from previous experiences
Adaptive capacity																		Education
Adaptive capacity																		Inter-organizational coordination
**Indicator A**	**9**	**8**	**7**	**6**	**5**	**4**	**3**	**2**	**1**	**2**	**3**	**4**	**5**	**6**	**7**	**8**	**9**	**Indicator B**
Planning strategy and goal setting																		Responsibility
Planning strategy and goal setting																		Readiness
Planning strategy and goal setting																		Resources
Planning strategy and goal setting																		Communication and teamwork

Planning strategy and goal setting																		Effective public participation
Planning strategy and goal setting																		Awareness of the situation
Planning strategy and goal setting																		Leadership and management
Planning strategy and goal setting																		Creativity and innovation
Planning strategy and goal setting																		Fallibility culture
Planning strategy and goal setting																		Learning from previous experiences
Planning strategy and goal setting																		Education
Planning strategy and goal setting																		Inter-organizational coordination
**Indicator A**	**9**	**8**	**7**	**6**	**5**	**4**	**3**	**2**	**1**	**2**	**3**	**4**	**5**	**6**	**7**	**8**	**9**	**Indicator B**
Readiness																		Resources
Readiness																		Communication and teamwork
Readiness																		Effective public participation
Readiness																		Awareness of the situation
Readiness																		Leadership and management
Readiness																		Creativity and innovation
Readiness																		Fallibility culture
Readiness																		Learning from previous experiences
Readiness																		Education
Readiness																		Inter-organizational coordination
**Indicator A**	**9**	**8**	**7**	**6**	**5**	**4**	**3**	**2**	**1**	**2**	**3**	**4**	**5**	**6**	**7**	**8**	**9**	**Indicator B**
Resources																		Communication and teamwork
Resources																		Effective public participation
Resources																		Awareness of the situation
Resources																		Leadership and management
Resources																		Creativity and innovation
Resources																		Fallibility culture
Resources																		Learning from previous experiences
Resources																		Education
Resources																		Inter-organizational coordination
**Indicator A**	**9**	**8**	**7**	**6**	**5**	**4**	**3**	**2**	**1**	**2**	**3**	**4**	**5**	**6**	**7**	**8**	**9**	**Indicator B**
Effective public participation																		Awareness of the situation
Effective public participation																		Leadership and management
Effective public participation																		Creativity and innovation
Effective public participation																		Fallibility culture
Effective public participation																		Learning from previous experiences
Effective public participation																		Education

Effective public participation																		Inter-organizational coordination
**Indicator A**	**9**	**8**	**7**	**6**	**5**	**4**	**3**	**2**	**1**	**2**	**3**	**4**	**5**	**6**	**7**	**8**	**9**	**Indicator B**
Leadership and management																		Creativity and innovation
Leadership and management																		Fallibility culture
Leadership and management																		Learning from previous experiences
Leadership and management																		Education
Leadership and management																		Inter-organizational coordination
**Indicator A**	**9**	**8**	**7**	**6**	**5**	**4**	**3**	**2**	**1**	**2**	**3**	**4**	**5**	**6**	**7**	**8**	**9**	**Indicator B**
Creativity and innovation																		Fallibility culture
Creativity and innovation																		Learning from previous experiences
Creativity and innovation																		Education
Creativity and innovation																		Inter-organizational coordination
**Indicator A**	**9**	**8**	**7**	**6**	**5**	**4**	**3**	**2**	**1**	**2**	**3**	**4**	**5**	**6**	**7**	**8**	**9**	**Indicator B**
Fallibility culture																		Learning from previous experiences
Fallibility culture																		Education
Fallibility culture																		Inter-organizational coordination
**Indicator A**	**9**	**8**	**7**	**6**	**5**	**4**	**3**	**2**	**1**	**2**	**3**	**4**	**5**	**6**	**7**	**8**	**9**	**Indicator B**
Learning from previous experiences																		Education
Learning from previous experiences																		Inter-organizational coordination
**Indicator A**	**9**	**8**	**7**	**6**	**5**	**4**	**3**	**2**	**1**	**2**	**3**	**4**	**5**	**6**	**7**	**8**	**9**	**Indicator B**
Education																		Inter-organizational coordination
